# Supplementary material for: Local parasite pressures and host genotype modulate epigenetic diversity in a mixed‐mating fish
Source: Ecol Evol. 2019 Jul 15;9(15):8736–48. doi: 10.1002/ece3.5426 (PMC6686343; doi:10.1002/ece3.5426)
Supplement: Supplementary file 1 [file ECE3-9-8736-s001.docx]

**Supplementary material for**

**Local parasite pressures and host genotype may modulate epigenetic diversity in a mixed-mating fish**

Waldir M. Berbel-Filho, Carlos Garcia de Leaniz, Paloma Morán, Jo Cable, Sergio M. Q. Lima and Sofia Consuegra

Supporting figures and tables


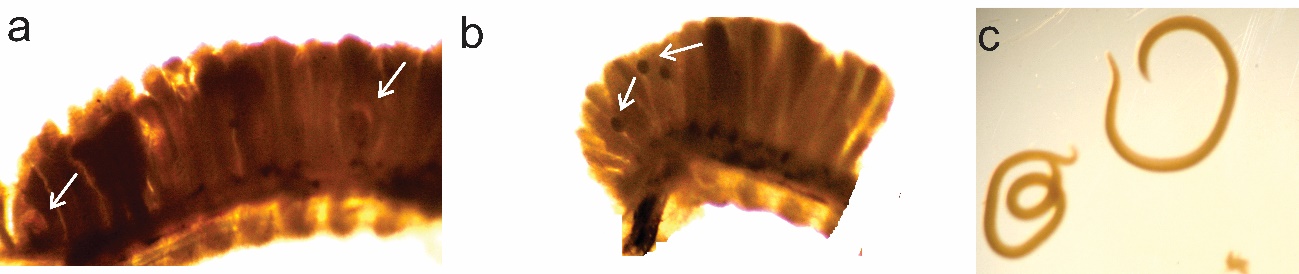


**Fig. S1** Parasites of *Kryptolebias hermaphroditus* from North-eastern Brazil: **a)** bacterial gill cyst; **b)** protozoan gill cysts; **c)** nematodes from the gut.


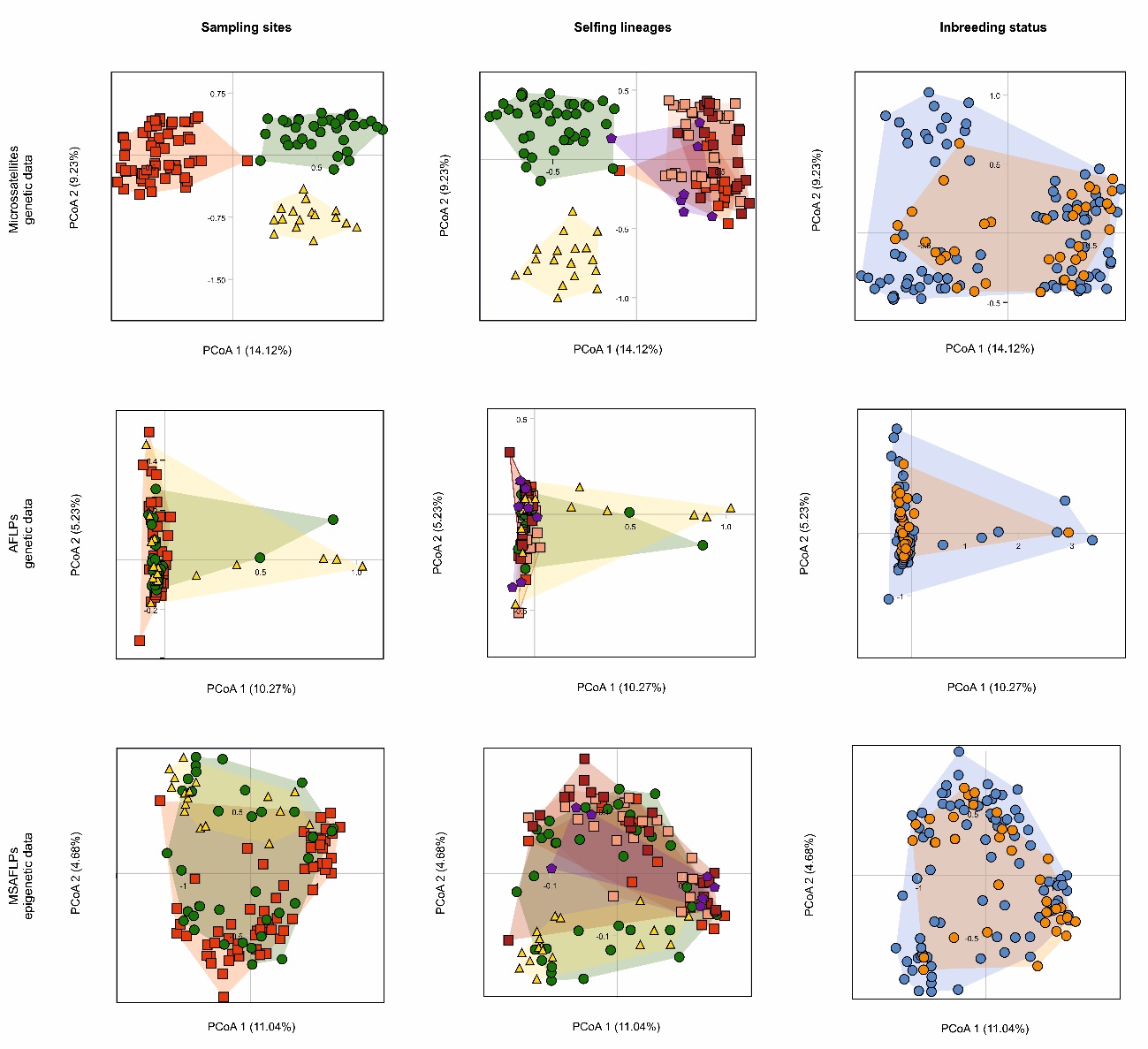


**Fig. S2** Principal coordinate analysis (PCoA) of genetic (microsatellites and AFLPs) and epigenetic (MSL) variation for sampling sites, selfing lineages identified by INSTRUCT and inbreeding status (selfed or outcrossed). Squares, circles and triangles represent site 1, site 2 and site 3, respectively. Lineages are coloured in red (1), salmon (2), green (3), brown (4) and yellow (5). Lineage 6 (shared between sites 1 and 2) was represented by purple pentagons. Blue and orange circles represented inbred and outcrossed individuals, respectively.


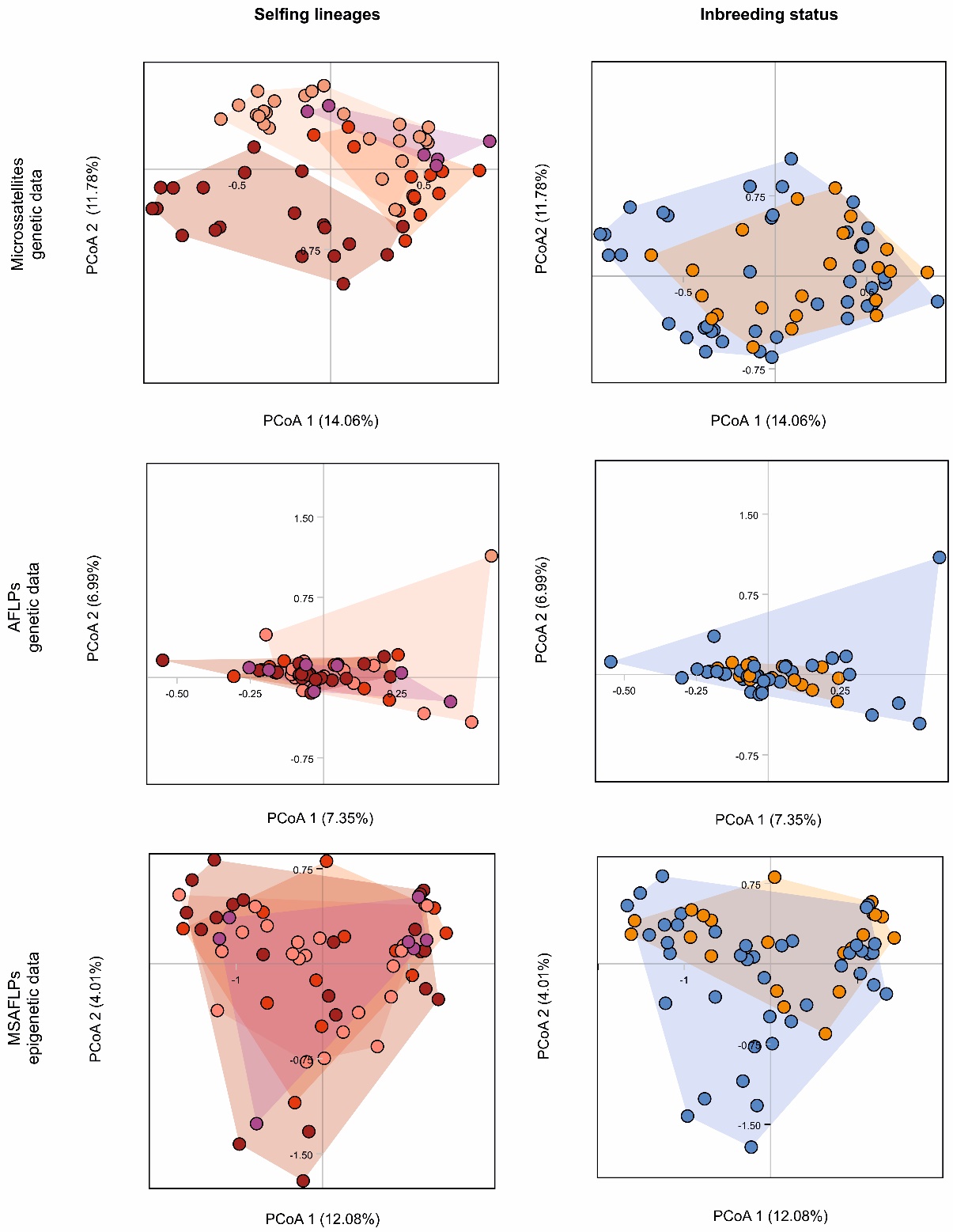


**Fig. S3** Principal coordinate analysis (PCoA) of genetic (microsatellites and AFLPs) and epigenetic (MSL) variation for selfing lineages identified by INSTRUCT and inbreeding status (selfed or outcrossed) for individuals in site 1. Filled circles with different colours represent the different selfing lineages from site 1 (red for lineage 1, salmon for lineage 2, brown for lineage 4, purple for lineage 6). Blue and orange circles represent inbred and outcrossed individuals, respectively

**Table S1** Genetic diversity (for 27 microsatellite loci), mean parasites number (standard deviation in brackets) and parasite prevalence in *Kryptolebias hermaphroditus* among selfing lineages identified by INTRUSCT in North-eastern Brazil. N= sampling size; N_a_ = mean number of alleles of alleles; H_e_ = expected heterozygosity; H_o_ = observed heterozygosity; F_IS_ = inbreeding coefficient; HL = homozygosity by locus; S = selfing rates.

|  | **Lineage 1** | **Lineage 2** | **Lineage 3** | **Lineage 4** | **Lineage 5** | **Linage 6** |
| --- | --- | --- | --- | --- | --- | --- |
| **N** | 14 | 25 | 41 | 22 | 18 | 8 |
| **Selfed/Outcrossed** | 7/7 | 17/8 | 29/12 | 15/7 | 16/2 | 8/0 |
| **Sampling site** | 1 | 1 | 2 | 1 | 3 | 1 (7) and 2 (1) |
| **Genetic diversity** |  |  |  |  |  |  |
| N_ma_ | 2.14 | 2.29 | 3.44 | 2.22 | 3.14 | 1.59 |
| H_e_ | 0.25 | 0.24 | 0.26 | 0.19 | 0.33 | 0.17 |
| H_o_ | 0.01 | 0.03 | 0.01 | 0.02 | 0.04 | 0.00 |
| F_IS_ | 0.97 | 0.85 | 0.89 | 0.88 | 0.87 | 0.97 |
| HL | 0.97 | 0.92 | 0.97 | 0.95 | 0.93 | 0.99 |
| S | 0.87 | 0.91 | 0.91 | 0.92 | 0.92 | 0.93 |
| **Parasite loads** |  |  |  |  |  |  |
| Bacterial gills cysts | 2.92 (2.18) | 2.24 (1.9) | 2.53 (3.02) | 3.13 (2.76) | 1.27 (0.80) | 7.12 (5.39) |
| Protozoan gills cysts | 0 | 0 | 1.56 (1.60) | 0 | 0.33 (1.37) | 0 |
| Nematodes | 0.07 (0.25) | 0.16 (0.46) | 0.02 (0.15) | 0.13 (0.62) | 0 | 0.37 (0.69) |
| Total parasite load | 3.14 (2.03) | 2.36 (1.85) | 4.12 (3.15) | 3.27 (3.01) | 1.61 (1.73) | 7.5 (5.54) |
| **Parasite prevalence (% of fish with infection)** |  |  |  |  |  |  |
| Bacterial gills cysts | 92.85 | 84.00 | 70.73 | 95.45 | 83.33 | 100.00 |
| Protozoan gills cysts | 0 | 0 | 41.46 | 0 | 5.55 | 0 |
| Nematodes | 7.14 | 16.00 | 2.43 | 4.54 | 0 | 25.00 |

**Table S2** Pairwise F_ST_ values among sampling sites and selfing lineages (k) in *Kryptolebias hermaphroditus*. Asterisks represent significance levels (***P ≤0.001).

| **Per sampling site** | | | | | | |
| --- | --- | --- | --- | --- | --- | --- |
|  | **1** | **2** | **3** |  |  |  |
| 1 | - |  |  |  |  |  |
| 2 | 0.25*** | - |  |  |  |  |
| 3 | 0.32*** | 0.28*** | - |  |  |  |
| **Per selfing lineage (k)** | | | | | | |
|  | **1** | **2** | **3** | **4** | **5** | **6** |
| 1 | - |  |  |  |  |  |
| 2 | 0.20*** | - |  |  |  |  |
| 3 | 0.34*** | 0.28*** | - |  |  |  |
| 4 | 0.27*** | 0.24*** | 0.35*** | - |  |  |
| 5 | 0.34*** | 0.36*** | 0.29*** | 0.40*** | - |  |
| 6 | 0.30*** | 0.28*** | 0.36*** | 0.40*** | 0.36*** | - |

**Table S3** Hierarchical analysis of molecular variance partitioning (AMOVA) for MS-AFLPs data among original and replicated samples to test for reproducibility. df= degrees of freedom; SSD= sum of squared deviations; Mol. var. (%) = molecular variance percentages from variance components sigma 2; ɸ_ST_ = Phi statistics for population differentiation. *P* value derived from 10,000 permutations.

|  | **df** | **SSD** | **Mol. var. (%)** | **ɸ_ST_** | ***P* value** |
| --- | --- | --- | --- | --- | --- |
| **Epigenetic data (MSL)** |  |  | | | |
| Between original and replicate | 1 | 30.48 | 0.19 | 0.002 | 0.53 |
| Within original and replicate | 46 | 1474 | 99.81 |  |  |
| **AFLP genetic data (NML)** |  |  | | | |
| Between original and replicate | 1 | 1.35 | 1.00 | 0.01 | 0.94 |
| Within original and replicate | 46 | 91.46 | 99.00 |  |  |

**Table S4** Hierarchical analysis of molecular variance (AMOVA) for microsatellites and MS-AFLPs data **a)** among selfing lineages identified by INSTRUCT and **b)** between inbreeding status (selfed and outcrossed) in *Kryptolebias hermaphroditus* for site 1 (Ceará-Mirim mangrove). df= degrees of freedom; Mol. var. (%) = molecular variance percentages from variance components sigma 2; *P* value derived from 10,000 permutations.

|  | **Microsatellites** | | | | **NML** | | | | **MSL** | | | | |
| --- | --- | --- | --- | --- | --- | --- | --- | --- | --- | --- | --- | --- | --- |
|  | df | Mol. var. (%) | F_ST_ | *P* value | df | Mol. var. (%) | ɸ_ST_ | *P* value | df | Mol. var. (%) | ɸ_ST_ | *P* value | |
| **a** Selfing lineages | | | | | | | | | | | | | |
| Among lineages | 3 | 27.91 | 0.27 | **0.001** | 3 | 0.83 | 0.008 | 0.12 | 3 | 0.15 | 0.006 | 0.52 | |
| Within lineages | 132 | 72.09 |  |  | 58 | 99.17 |  |  | 58 | 99.85 |  |  | |
| **b** Inbreeding status | | | | | | | | | | | | |  |
| Between selfed and outcrossed | 1 | 3.42 | 0.03 | **0.001** | 1 | 0.16 | 0.006 | 0.57 | 1 | 0.64 | 0.001 | 0.20 | |
| Within selfed and outcrossed | 134 | 96.58 |  |  | 60 | 99.84 |  |  | 60 | 99.36 |  |  | |

**Table S5** Results of the best generalized linear models indicated by the multi-model averaging approach for the proportion of methylated loci in *Kryptolebias hermaphroditus*. Models are ranked according to their corrected Akaike Information Criterion (AICc), the distance between a given model and the best fitting model (ΔAICc), the Akaike weight (Wi), and the evidence ratio (ER), which represents the ratio between the weights of the best and competing models. Only the best-fitting models within two AICc units are shown.

| **Independent variable** | **df** | **z-value** | ***P*-value** | **AICc** | **ΔAICc** | **W_i_** | **ER** |
| --- | --- | --- | --- | --- | --- | --- | --- |
| **Proportion of methylated loci** | | | | | | | |
| ***Model 1*** |  |  |  | 1065.5 | 0.00 | 0.28 | 1.00 |
| Selfing lineage | 5 | -4.50 | **<0.001** |  |  |  |  |
| Scaled parasite load | 1 | -0.02 | 0.83 |  |  |  |  |
| Inbreeding | 1 | 1.73 | 0.15 |  |  |  |  |
| Selfing lineage x parasite scaled | 5 | -3.90 | **0.005** |  |  |  |  |
| Selfing lineage x inbreeding | 4 | -1.64 | **0.04** |  |  |  |  |
| ***Model 2*** |  |  |  | 1066.5 | 1.00 | 0.17 | 1.39 |
| Selfing lineage | 5 | -6.48 | **<0.001** |  |  |  |  |
| Inbreeding | 1 | -2.53 | 0.15 |  |  |  |  |
| Selfing lineage x inbreeding | 4 | -2.22 | 0.04 |  |  |  |  |

**Table S6** Results of the best generalized linear models indicated by the multi-model averaging approach for the proportion of methylated loci in *Kryptolebias hermaphroditus* (including homozygosity index as independent variable). Models are ranked according to their corrected Akaike Information Criterion (AICc), the distance between a given model and the best fitting model (ΔAICc), the Akaike weight (Wi), and the evidence ratio (ER), which represents the ratio between the weights of the best and competing models. Only the best-fitting models within two AICc units are shown.

| **Independent variable** | **df** | **z-value** | ***P*-value** | **AICc** | **ΔAICc** | **W_i_** | **ER** |
| --- | --- | --- | --- | --- | --- | --- | --- |
| **Proportion of methylated loci** | | | | | | | |
| ***Model 1*** |  |  |  | 1021.9 | 0.00 | 0.04 | 1.00 |
| Selfing lineage | 5 | -6.50 | **<0.001** |  |  |  |  |
| Scaled parasite load | 1 | -0.21 | 0.83 |  |  |  |  |
| Inbreeding | 1 | -1.19 | **0.05** |  |  |  |  |
| Homozygosity (HL) | 1 | -0.32 | 0.29 |  |  |  |  |
| Scaled parasite load x HL | 1 | 3.17 | 0.28 |  |  |  |  |
| Inbreeding x HL | 5 | 7.22 | **<0.001** |  |  |  |  |
| Selfing lineage x HL | 1 | 12.13 | **<0.001** |  |  |  |  |
| ***Model 2*** |  |  |  | 1023.2 | 1.30 | 0.02 | 3.19 |
| Selfing lineage | 5 | -5.70 | **<0.001** |  |  |  |  |
| Scaled parasite load | 1 | -0.37 | 0.83 |  |  |  |  |
| Inbreeding | 1 | -1.19 | 0.05 |  |  |  |  |
| Homozygosity (HL) | 1 | -0.35 | 0.29 |  |  |  |  |
| Scaled parasite load x HL | 1 | 3.14 | 0.10 |  |  |  |  |
| Inbreeding x HL | 5 | 6.52 | **<0.001** |  |  |  |  |
| Selfing lineage x HL | 1 | 11.22 | **<0.001** |  |  |  |  |
| Selfing lineage x Inbreeding | 4 | -3.10 | **0.01** |  |  |  |  |

**Table S7** Results of the best generalized linear model indicated by the multi-model averaging approach for the proportion of methylated loci including the number of bacterial cysts as a predictor in *Kryptolebias hermaphroditus*. Models are ranked according to their corrected Akaike Information Criterion (AICc), the distance between a given model and the best fitting model (ΔAICc), the Akaike weight (W_i_), and the evidence ratio (ER), which represents the ratio between the weights of the best and competing models. Only the best-fitting models within two AICc units are shown.

| **Independent variable** | **df** | **z-value** | ***P*-value** | **AICc** | **ΔAICc** | **W_i_** | **ER** |
| --- | --- | --- | --- | --- | --- | --- | --- |
| **Proportion of methylated loci (with number of bacterial cysts as a predictor)** | | | | | | | |
| ***Model 1*** |  |  |  | 1060.8 | 0.00 | 0.51 | 1.00 |
| Selfing lineage | 5 | -9.23 | **<0.001** |  |  |  |  |
| Bacterial cysts | 1 | -2.93 | 0.88 |  |  |  |  |
| Inbreeding | 1 | -1.17 | 0.15 |  |  |  |  |
| Selfing lineage x Bacterial cysts | 5 | 9.09 | **<0.001** |  |  |  |  |
| Selfing lineage x inbreeding | 4 | -4.97 | **0.04** |  |  |  |  |

**Table S8** Results of the best generalized linear modes indicated by the multi-model averaging approach for the proportion of methylated loci in *Kryptolebias hermaphroditus* sampled on sampling site 1. Models are ranked according to their corrected Akaike Information Criterion (AICc), the distance between a given model and the best fitting model (ΔAICc), the Akaike weight (W_i_), and the evidence ratio (ER), which represents the ratio between the weights of the best and competing models. Only the best-fitting models within two AICc units are s6hown.

| **Independent variable** | **df** | **z/t-value** | ***P*-value** | **AICc** | **ΔAICc** | **W_i_** | **ER** |
| --- | --- | --- | --- | --- | --- | --- | --- |
| **Proportion of methylated loci** | | | | | | | |
| ***Model 1*** |  |  |  | 621.86 | 0.00 | 0.29 | 1.00 |
| Selfing lineage | 3 | -7.09 | **0.04** |  |  |  |  |
| Scaled parasite load | 1 | -5.02 | 0.31 |  |  |  |  |
| Inbreeding | 1 | -3.95 | **0.04** |  |  |  |  |
| Inbreeding x parasite scaled | 1 | -10.43 | **0.01** |  |  |  |  |
| Selfing lineage x inbreeding | 2 | -9.52 | **0.001** |  |  |  |  |
| ***Model 2*** |  |  |  | 621.88 | 0.02 | 0.29 | -0.66 |
| Scaled parasite load | 1 | -11.49 | **0.03** |  |  |  |  |
| Inbreeding | 1 | -10.64 | **0.09** |  |  |  |  |
| Inbreeding x scaled parasite load | 1 | -17.93 | **<0.001** |  |  |  |  |
| ***Model 3*** |  |  |  | 622.70 | 0.82 | 0.19 | 1.82 |
| Selfing lineage | 3 | -3.61 | **0.04** |  |  |  |  |
| Scaled parasite load | 1 | -3.81 | 0.31 |  |  |  |  |
| Inbreeding | 1 | -2.15 | **0.04** |  |  |  |  |
| Inbreeding x parasite scaled | 1 | -7.04 | **0.01** |  |  |  |  |
| Selfing lineage x inbreeding | 2 | -7.10 | **0.01** |  |  |  |  |
| Selfing lineage x parasite scaled | 3 | -6.50 | **0.005** |  |  |  |  |
| ***Model 4*** |  |  |  | 623.74 | 1.88 | 0.11 | 1.67 |
| Selfing lineage | 3 | -10.72 | **0.04** |  |  |  |  |
| Scaled parasite load | 1 | -5.88 | 0.31 |  |  |  |  |
| Inbreeding | 1 | -4.08 | **0.04** |  |  |  |  |
| Inbreeding x parasite scaled | 1 | -11.39 | **<0.001** |  |  |  |  |
